# Identification and characterization of telocytes in the uterus of the oviduct in the Chinese soft-shelled turtle, *Pelodiscus sinensis*: TEM evidence

**DOI:** 10.1111/jcmm.12392

**Published:** 2014-09-18

**Authors:** Shakeeb Ullah, Ping Yang, Linli Zhang, Qian Zhang, Yi Liu, Wei Chen, Yasir Waqas, Yuan Le, Bing Chen, Qiusheng Chen

**Affiliations:** Key Laboratory of Animal Physiology and Biochemistry, Ministry of Agriculture, College of Veterinary Medicine, Nanjing Agricultural UniversityNanjing, China

**Keywords:** telocytes, uterus, Chinese soft-shelled turtle (*Pelodiscus sinensis)*, ultrastructure

## Abstract

Telocytes (Tcs) are cells with telopodes (Tps), which are very long cellular extensions with alternating thin segments (podomers) and dilated bead-like thick regions known as podoms. Tcs are a distinct category of interstitial cells and have been identified in many mammalian organs including heart, lung and kidney. The present study investigates the existence, ultrastructure, distribution and contacts of Tcs with surrounding cells in the uterus (shell gland) of the oviduct of the Chinese soft-shelled turtle, *Pelodiscus sinensis*. Samples from the uterine segment of the oviduct were examined by transmission electron microscopy. Tcs were mainly located in the lamina propria beneath the simple columnar epithelium of the uterus and were situated close to nerve endings, capillaries, collagen fibres and secretory glands. The complete morphology of Tcs and Tps was clearly observed and our data confirmed the existence of Tcs in the uterus of the Chinese soft-shelled turtle *Pelodiscus sinensis*. Our results suggest these cells contribute to the function of the secretory glands and contraction of the uterus.

## Introduction

Telocytes (TCs) are a novel type of interstitial (stromal) cell that have been recently described in humans and other mammals in both cavity and non-cavity organs [1]. TCs are cells that have very long cellular extensions termed telopodes (Tps; tens to hundreds of micrometers) with alternating thin segments known as podomers (∼70 nm, which is below the resolving power of light microscopy) and dilated bead-like thick regions (podoms). These structures accommodate a large number of mitochondria, endoplasmic reticulum and caveolae, and can secrete exosomes [2]. Tps are the most characteristic features of TCs. These are thin, long and moniliform in structure and sometimes with a convoluted trajectory. These features make TCs different from other stromal cell types such as fibrocytes, fibroblasts, or fibroblast-like cells [3]. TCs initially appeared similar to interstitial cells described by Cajal (ICCs), because they appeared to have ultrastructural features of ICCs. However, it was soon confirmed that they were different cells and that TCs had a different ultrastructure than ICCs [3]. As a result, the cells that were initially described as interstitial Cajal-like cells (ICLCs) were renamed as TCs, and their long prolongations were called Tps [1,3]. TCs have been identified in the interstitial space of the following major organs: heart [4], parotid glands [5], intestine [6], lungs [7,8], trachea [7,9], pleura [10], skeletal muscle [11], pericardium [4], pulmonary veins [12], mesentery [13], gall bladder [14], exocrine pancreas [15,16], mammary gland [17,18], placenta [19,20], blood vessels [21,22], female reproductive duct, uterus, fallopian tube [23,24], myometrium [25,26] and endometrium [27].

Various functions of TCs are possible based on their morphological characteristics. In some organs, TCs act as supporting cells for tissue organization [28,29]. TCs can also receive/generate molecular signals from/to other cells that can influence the nearby myocytes by juxta/paracrine mechanisms [30,31]. As a specific functional characteristic, TCs have a major role in intercellular signalling over both short and long distances. The long Tps form direct contacts (junctions) with neighbouring cells and contribute to the (directional) transport of long-range signals driven by TCs [32,33]. However, local (paracrine) signalling of TCs is achieved by shedding vesicles [15,33,34]. It has been proposed that TCs play vital roles in angiogenesis and TCs are frequently found close to small vessels and express angiogenesis-related factors (VEGF, NO) and pro-angiogenic microRNAs [34]. TCs, leucocytes and myocytes work together to maintain pregnancy or induce labour [26]. CD117-positive cells that are similar to TCs prevent untimely uterine contraction during the mid-gestational period [35]. The importance of TCs in pathology cannot be ignored, and it was recently suggested that they might represent the common cell of origin of various stromal tumours because they generate extra-gastrointestinal stromal tumours and perivascular epithelioid cell tumours [36,37]. The ultrastructural composition of TCs has been complemented with immunophenotypical and electrophysiological characterizations. Additionally, the specific microRNA expression signature has been elucidated [34,38,39]. TCs are different from mesenchymal stem cells and fibroblasts. The gene signature of TCs suggests specific biological functions in development and tissue morphogenesis, biological compound transport and extracellular matrix remodelling [40]. Recently, a quantitative proteomics approach identified numerous proteins from TCs. The protein expression profile showed many up-regulated proteins including myosin-14 and periplakin, which suggests that TCs might play distinct roles in mechanical sensing and mechanochemical conversion tasks, tissue homoeostasis and remodelling/renewal. Additionally, the up-regulated proteins match those found in extracellular vesicles and this finding highlights the role of TCs in intercellular signalling and stem cell niche modulation [41].

Most studies of TCs have been performed in mammalian species and there are no studies available in the literature examining TCs in reptiles. The reptilian oviduct is an important organ that performs major functions including sperm storage, egg formation, maturation and transportation of unfertilized or fertilized eggs to the exterior of the body. The organ is composed of the following five segments or regions (from anterior to posterior): infundibulum, magnum, isthmus, shell gland (uterus) and vagina. The uterus is an important segment of the oviduct and has several important functions. In addition to salt and water absorption, egg calcification occurs in the uterus to form the calcium carbonate shell. Sperm storage also takes place in the uterus in some reptile species. The egg pigmentation also occurs and finally the uterus (shell gland) contracts to expel eggs. Egg transport is accomplished mainly by contractions of the oviduct, and the oviduct musculature functions as a stretch receptor. The ovum also provides mechanical stimulus by itself [42,43]. Similar to other smooth muscle organs, the reptile oviduct undergoes a specific pattern of electrical and mechanical activity during egg passage. It has been shown in previous studies that there are variable intermittent high-rise or frequent electrical potentials before and around egg transport in different segments of the oviduct in birds [44]. These electrical waves are ultimately responsible for the physiological contractions of the uterine tube.

The present work was performed with the uterus (shell gland) and oviduct of the Chinese soft-shelled turtle *Pelodiscus sinensis*, by utilizing transmission electron microscopy. The Chinese soft-shelled turtle *P. sinensis* is an ancient species of reptiles distributed throughout China and many other countries. This species has considerable importance in aquaculture and is an important food source. The aim of this study is to identify TCs and investigate their morphological and ultrastructural characteristics. We have also examined their contact with the surrounding cells in the uterus of the oviduct.

## Materials and methods

### Animals

Ten female adult soft-shelled turtles, *P. sinensis*, were purchased from a wild breeding base in Jiangsu province of China. The animals each weighed 660–720 g. The animals were anesthetized by sodium pentobarbital (20 mg/kg) administered intraperitoneally and were killed by cervical dislocation. The uterus samples were collected for transmission electron microscopy. All protocols were approved by Science and Technology Agency of Jiangsu Province (SYXK (SU) 2010-0005).

### Transmission electron microscopy

The samples were cut into small blocks and then fixed in 2.5% glutaraldehyde in PBS (4°C, pH 7.4, 0.1 M) for 24 hrs. The blocks were rinsed in the same PBS and then post-fixed for 60 min. at room temperature in the same way by using buffered 1% osmium tetroxide (Polysciences Inc. Warrington, PA, USA) and washed in the buffer. The samples were then dehydrated in ascending concentrations of ethyl alcohol, infiltrated with a propylene oxide–Araldite mixture and then embedded in Araldite. The blocks were then sectioned by using an ultramicrotome (Reichert-Jung, Wien, Austria) and the ultrathin sections (50 nm) were mounted on formvar-coated grids. The sections were stained with 1% uranyl acetate and Reynold's lead citrate for 20 min. The sections were examined and photographed by using a high resolution digital camera (16 mega pixel) connected to the TEM, Hitachi H-7650 (Japan).

## Results

**Fig. 1:**
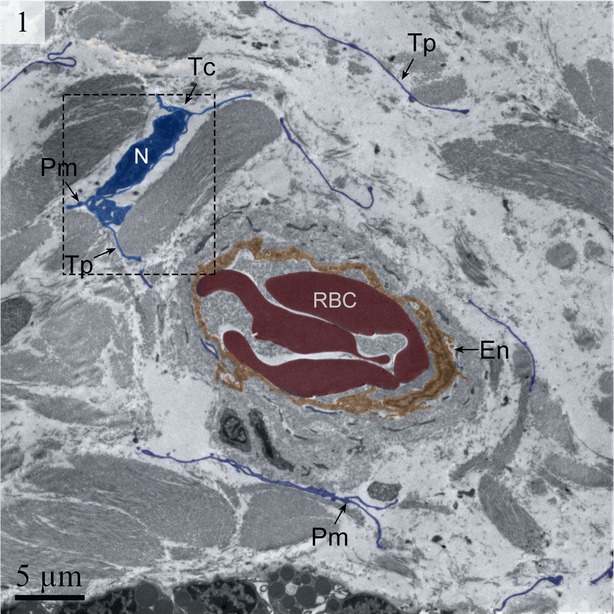
Digitally coloured TEM image of the turtle uterus. Long telopodes are present around the capillary. The podom and a telocyte are also visible. (Tc, telocyte; Tp, telopode; RBC, red blood cell; En, endothelium). The scale bar represents 5 μm.

**Fig. 2:**
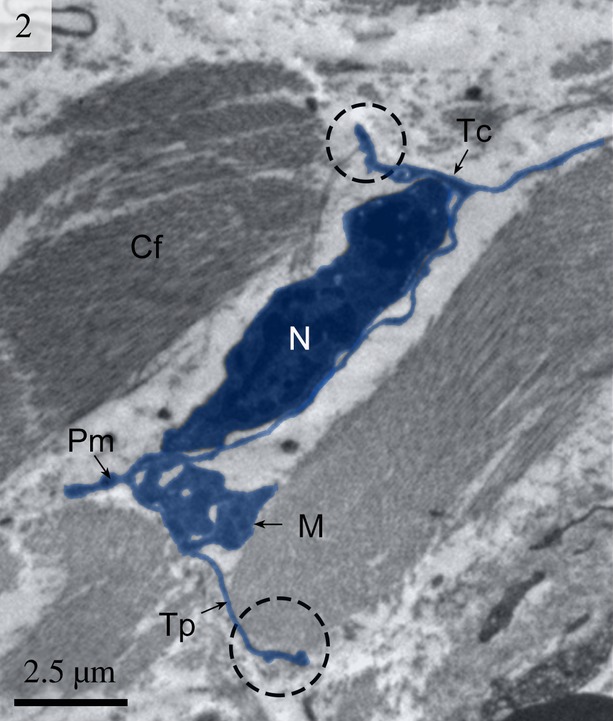
Digitally coloured TEM image of the turtle uterus (enlarged rectangular area of Fig. 1) showing a telocyte with its long telopodes. Circular areas show the close connection of telopodes with collagen fibres. The telopode is very long with alternating podom and podomer. The mitochondria are also visible. (Tc, telocyte; Tp, telopode; Pd, podomer; N, nucleus; M, mitochondria; Cf, collagen fibres). The scale bar represents 2.5 μm.

**Fig. 3:**
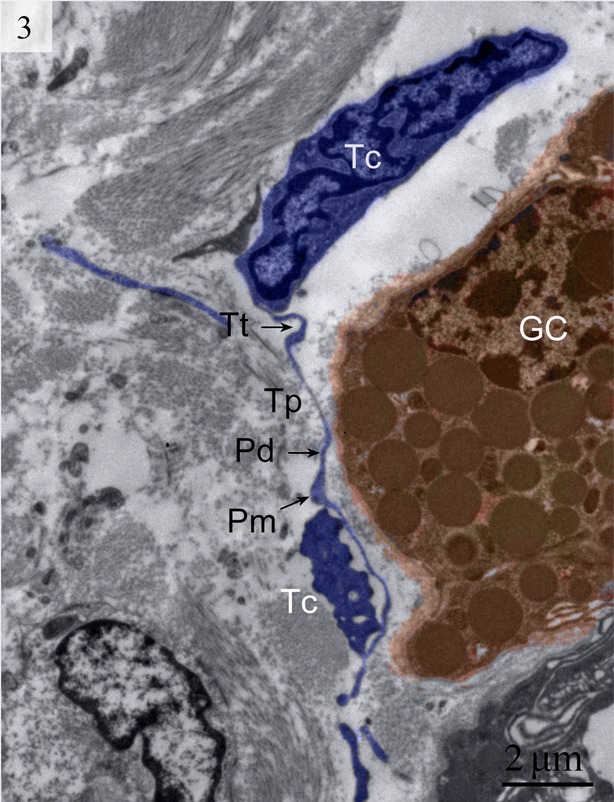
Digitally coloured TEM image of the turtle uterus. At least 2 telocytes with their extensive telopodes are visible in the lamina propria near gland cells. Telopodes with long alternating podom and podomer are visible. (Tt, tortuous telopode; Tc, telocytes; GC, gland cells; Pm, podom; Pd, podomer). The scale bar represents 2 μm.

**Fig. 4:**
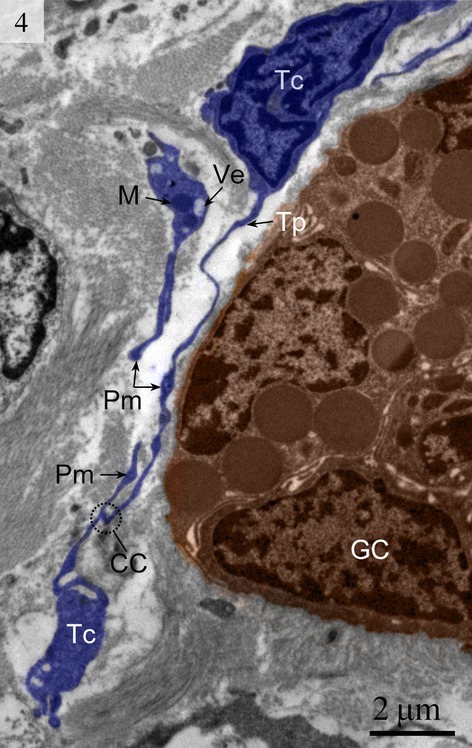
Digitally coloured TEM image of the turtle uterus. Two telocytes are clearly visible and telocytes connect with telopodes from other telocytes. The encircled area shows the close connection between telopodes. Mitochondria and vesicles are also visible. (TC, telocyte; Tp, telopode; GC, gland cell; CC, close connection; Ve, vesicle; M, mitochondria). The scale bar represents 2 μm.

Telocytes have Tps containing typical podoms that were easy to recognize in our TEM images in the lamina propria (Figs 1 and 2) beneath the simple columnar epithelium of the uterus. These cells meet the ultrastructural criteria for TCs (Fig. 2) and have long (tens of micrometers) and thin (50–200 nm) cellular processes called Tps. The prolongations emerge from the cell bodies and are very long and extremely twisted. The dilated part of the prolongation is clearly visible (Fig. 3). There are mitochondria (Figs 2 and 4) and vesicles in both the cell bodies and the processes (Fig. 4).

**Fig. 5:**
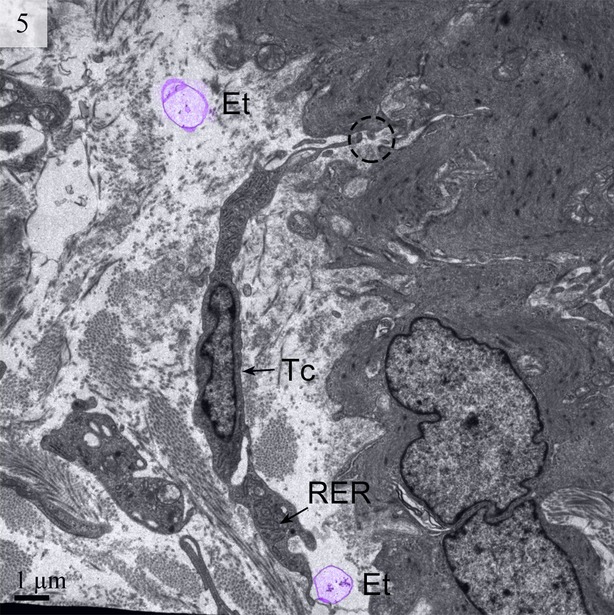
TEM image of the turtle uterus. The encircled area shows the close connection of telopode with smooth muscle. Ectosomes (digitally coloured violet) and rough endoplasmic reticulum are visible. (Tc, telocyte; RER, rough endoplasmic reticulum; Et, ectosomes). The scale bar represents 1 μm.

The absence of TC basal lamina was noted. Other organelles, such as the Golgi body and ribosomes, were rarely seen in our images. However, rough endoplasmic reticulum could be clearly seen (Fig. 5).

**Fig. 6:**
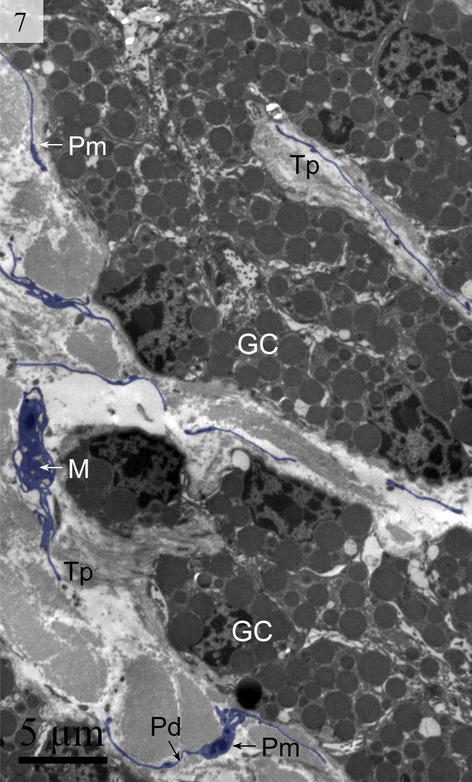
Digitally coloured TEM image of the turtle uterus. The telocyte and telopode exist in the uterine lamina propria in close proximity to gland cells and form a labyrinthine structure. Gland cells, telopodes, podom and podomer are visible. (M, mitochondria; Pm, podom; Tp, telopode; Pd, podomer; GC, gland cells). The scale bar represents 5 μm.

**Fig. 7:**
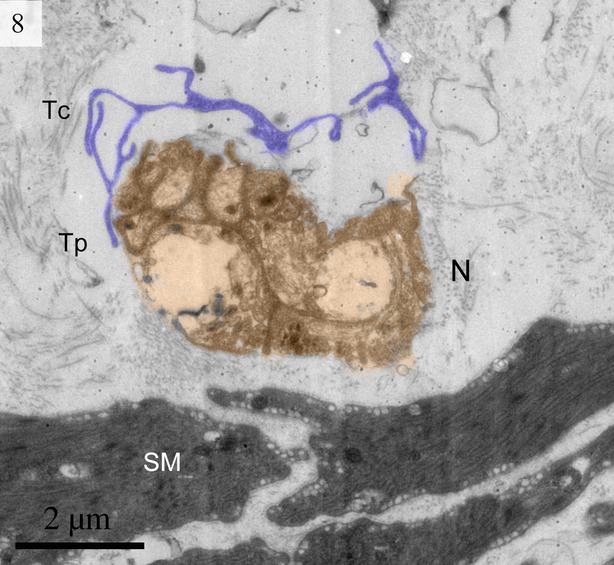
Digitally coloured TEM image of the turtle uterus. A telocyte is visible in close proximity to a nerve. (N, nerve; Tc, telocyte; Tp, telopodes; SM, smooth muscle). The scale bar represents 2 μm.

We detected Tps as discontinuous segments with alternating podom and podomer. The Tps were present near the blood vessels and there were network structures or labyrinthine structures between Tps (Figs 1 and 6). We also found Tps in close proximity to secretory gland cells in the lamina propria (Figs 3 and 6). The nuclei of TCs contained clusters of heterochromatin attached to the nuclear envelope and they appeared in irregular shapes. The TCs were linked with smooth muscles (Fig. 5) and nerve endings (Fig. 7) by their long Tps. The TCs were also connected with each other through their Tps (Fig. 4).

**Fig. 8:**
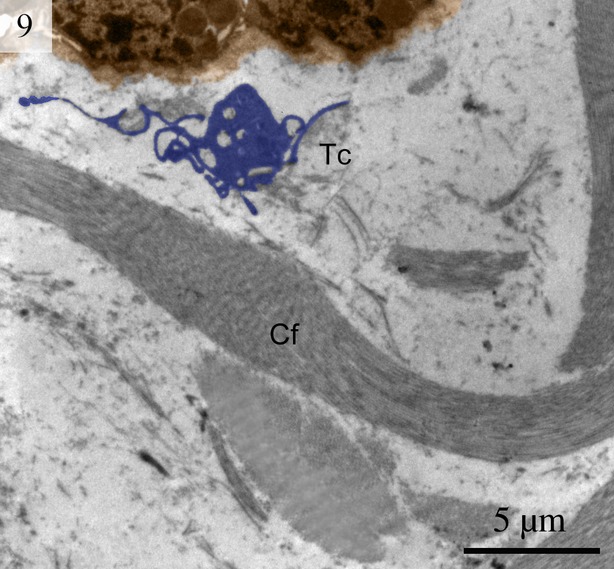
Digitally coloured TEM image of the turtle uterus. A telocyte with a convoluted telopode can be seen near the gland cells and collagen fibres. (Tc, telocyte; Cf, collagen fibres). The scale bar represents 5 μm.

**Fig. 9:**
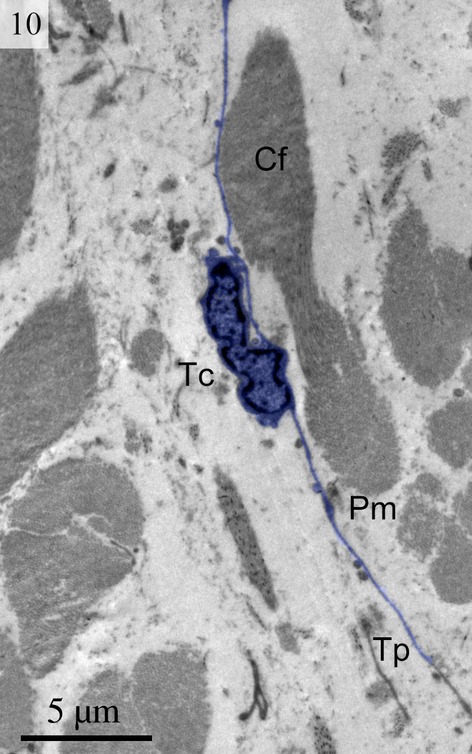
Digitally coloured TEM image of the turtle uterus. A telocyte with long thin telopode and podom is clearly visible near to the collagen firers. (Tc, telocyte; Tp, telopode; Pm, podom; Cf, collagen fibres). The scale bar represents 5 μm.

**Fig. 10:**
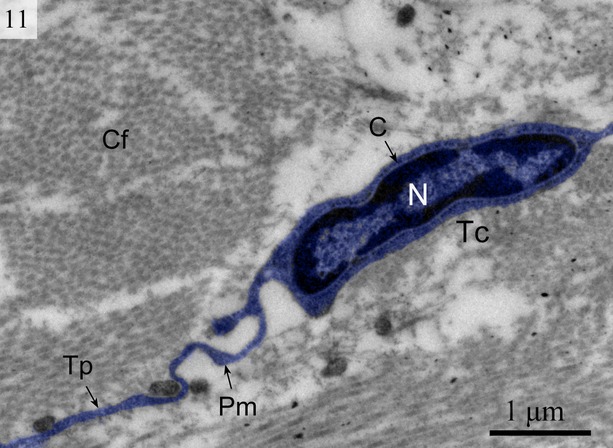
Digitally coloured TEM image of the turtle uterus showing typical telocyte and telopodes connected with collagen fibres. (N, nucleus; C, cytoplasm; Tp, telopode; Pm, podom). The scale bar represents 1 μm.

**Fig. 11:**
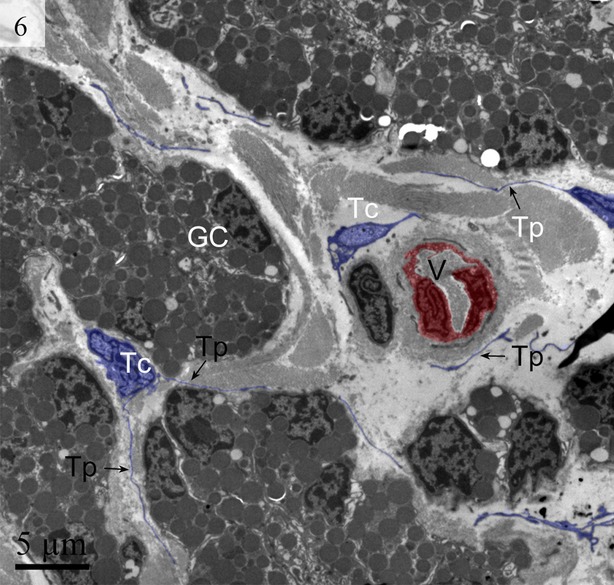
Digitally coloured TEM image of the turtle uterus. Telocytes with long telopode are visible around the gland cells and blood vessel form a labyrinthine structure. Gland cells, telocytes, telopodes are prominent. (GC, gland cells; Tc, telocyte; Tp, telopode). The scale bar represents 5 μm.

We found TCs were closely connected with collagen fibres in cross and longitudinal sections (Figs 2, 8, 9 and 10). Additionally, the TCs had different shapes (spindle to triangular) depending on the number of prolongations (Figs. 2, 3, 8 and 11). There were distinctive ectosomes found near the TCs within the longitudinal and circular muscle layer (Fig. 5).

## Discussion

This is the first study to identify TCs in animals besides mammals. We have demonstrated the morphological and ultrastructural characteristics of these cells in the uterus (shell gland) of the oviduct of Chinese soft-shelled turtle, *P. sinensis* by TEM. Electron microscopy is currently critical for accurate identification of TCs [1,3,11]. However, care should be taken in TEM studies when differentiating TCs from other stromal cell types, such as fibroblasts. Fibroblasts can be differentiated from TCs on the basis of their short cell processes with thick protrusions from the cell body. Furthermore, they have a different phenotype from Tps, which are long, moniliform and convoluted [3]. Our data clearly show the existence of long, moniliform and convoluted Tps. A comparative proteomic analysis of human lung TCs *versus* fibroblasts showed that TCs are different from fibroblasts [41]. We found that TCs connect different cell types by their long cell processes and are able to carry signals over long distances [32]. Conversely, local (paracrine) signalling of TCs is achieved by shedding vesicles [15,34,38].

The general features of the TCs we identified in the interstitial spaces of the oviduct of soft-shelled turtle *P. sinensis* are similar to those already reported in mammalian species and are consistent with the diagnostic criteria of TCs [1]. The TEM images showed TCs display a slender shaped cell body with a thin rim of cytoplasm surrounding the nucleus and extremely long and thin tubular processes. These Tps (up to 100 micrometers long, yet only 20–200 nanometers wide) emerge from the cell body. Tps consist of long thin tubes (podomers) interspersed with short dilations (podoms). The podoms contain abundant mitochondria and endoplasmic reticulum [26,45]. Our data indicate that ∼2–3 Tps were observed in a single section depending on the angle and site of the section. It was difficult to see 3-dimensional convolutions of the telopode for the full length in a section [24]. The close contact between TCs, nerve fibres and capillaries has been demonstrated [46]. Our findings are consistent with these results.

Collagen-embedded TCs were found in the dermis [29], which suggests that TCs might be involved in homoeostasis, remodelling, regeneration and skin repair [47]. The results of the present study are consistent with this view because TCs were found in close contact with collagen fibres. We also found that Tps connect with each other by either end-to-end or side-to-side contacts, but rarely by end-to-side connections. These data provide morphological evidence for the presumption that Tps might convey signals or have unique communication between TCs [20]. TCs are involved in intercellular signalling because of their strategic position near other cells such as nerve endings, capillaries and the 3-dimensional network of Tps [6]. It has been suggested that TCs also play roles in the nervous system, vascular system, immune system, interstitium, stem cells/progenitors and cardiomyocytes [38]. TCs form a dense, convoluted network linking TCs with each other and to other cells and tissues, including secretory acini and exocrine epithelial ducts, nerve fibres, macrophages and blood vessels. The results of the present study are consistent with these findings. The direct contact of TCs with endothelial tubes and their indirect positive influence within angiogenic zones suggests there is significant participation of TCs in neo-angiogenesis during the late stage of myocardial infarction [34].

Previous reports indicate that Tps generally form and release vesicles (or exosomes) that help TCs participate in intercellular communication. As in the heart, heterocellular communication among TCs and cardiomyocytes occurs by shedding vesicles and close proximity [48]. Our findings are in agreement with these recent studies.

Intercellular signalling can take place by two mechanisms. These mechanisms include paracrine and/or juxtacrine secretion of small signalling molecules and the shedding of microvesicles. The microvesicles can transport ‘packets’ of macromolecules to the target cells and alter their physiology. These vesicles can transport RNA or DNA among neighbouring cells and induce epigenetic changes [49,50]. The results of the current study show the presence of ectosomes released by TCs within the longitudinal and circular muscle layer. Ectosomes are vesicles that bud directly from the cell surface. The major characteristics of ectosomes released by tumour cells, polymorphonuclear leucocytes and erythrocytes are the expression of phosphatidylserine and anti-inflammatory/immunosuppressive activities similar to apoptotic cells [51]. The term ‘ectocytosis’ is used for the release of ectosomes [52]. Although ectocytosis describes the same phenomenon in all cell types, the stimuli inducing cell-membrane budding can differ from cell to cell. Endothelial and circulating blood cells release ectosomes when exposed to specific stimuli such as complement proteins [53]. Many cancer cells have an activated phenotype with highly active ectocytosis in the absence of any stimulus [54,55]. Although the shedding of ectosomes is enhanced when cells are activated, ectocytosis is an ongoing process *in vivo* for many cells [56]. Ectosomes are involved in the down regulation of inflammation and immunity [51]. The functions of the cells are always closely associated with their structure and morphology and there are important functions for TCs in the oviduct of reptiles. Our results clearly demonstrate the presence of Tps in a close proximity to secretory glands in the lamina propria beneath the simple columnar epithelium. There are also TCs near the blood vessel layer. Furthermore, there are connections with smooth muscle fibres formed by gap junctions and connective tissues. This finding suggests that TCs could influence the timing of contractile activity in smooth muscle cells and indicate the key role of TCs in uterine contraction. We speculate that TCs in the uterus display rhythmic electrical activity in the initiation and propagation of uterine contraction [26,57,58]. In conclusion, we have clearly identified TCs and evaluated their morphological and ultrastructural characteristics in the interstitial spaces in the uterus of the oviduct of the soft-shelled turtle *P. sinensis*. Tps connect with each other and with smooth muscle cells, nerve fibres, blood vessels and collagen fibres. These data demonstrate the existence of TCs in reptiles. Further studies must explore the potential bio-functions of TCs in certain pathological conditions in the uterus and investigate the mechanisms of interaction between TCs and other cells.
